# Acute flaccid paralysis incidence and Zika virus surveillance, Pacific Islands

**DOI:** 10.2471/BLT.16.171892

**Published:** 2017-01-01

**Authors:** Adam T Craig, Michelle T Butler, Roberta Pastore, Beverley J Paterson, David N Durrheim

**Affiliations:** a School of Medicine and Public Health, University of Newcastle, University Drive, Callaghan 2308, NSW, Australia.; b Population Health, Hunter New England Health, Wallsend, Australia.; c Expanded Programme on Immunization, World Health Organization Regional Office for the Western Pacific, Manila, Philippines.

## Abstract

**Problem:**

The emergence of Zika virus has challenged outbreak surveillance systems in many at-risk, low-resource countries. As the virus has been linked with Guillain–Barré syndrome, routine data on the incidence of acute flaccid paralysis (AFP) may provide a useful early warning system for the emergence of Zika virus.

**Approach:**

We documented all Zika virus outbreaks and cases in 21 Pacific Islands and territories for the years 2007 to 2015. We extracted data from the Global Polio Eradication Initiative database on the reported and expected annual incidence of AFP in children younger than 15 years. Using a Poisson probability test, we tested the significance of unexpected increases in AFP in years correlating with Zika virus emergence. Data were analysed separately for each Pacific Island country and territory.

**Local setting:**

In most Pacific Island countries, early warning surveillance for acute public health threats such as Zika virus is hampered by poor health infrastructure, insufficient human resources and geographical isolation.

**Relevant changes:**

Only one example was found (Solomon Islands in 2015) of a significant increase in reported AFP cases correlating with Zika virus emergence.

**Lessons learnt:**

We found no conclusive evidence that routinely reported AFP incidence data in children were useful for detecting emergence of Zika virus in this setting. More evidence may be needed from adult populations, who are more likely to be affected by Guillain–Barré syndrome. Reporting of AFP may be deficient in regions certified as polio-free.

## Résumé

**Problème:**

L'émergence du virus Zika pose un problème pour les systèmes de surveillance des épidémies dans de nombreux pays à faibles ressources et à risque. Étant donné qu'un lien a été établi entre ce virus et le syndrome de Guillain-Barré, les données systématiquement collectées sur l'incidence de la paralysie flasque aiguë (PFA) pourraient peut-être aider à établir un système d'alerte anticipée pour l'émergence du virus Zika.

**Approche:**

Nous avons documenté toutes les flambées de virus Zika et tous les cas d'infection à virus Zika dans 21 îles et territoires du Pacifique, sur la période 2007 à 2015. À partir de la base de données de l'Initiative mondiale pour l’éradication de la poliomyélite, nous avons extrait des données sur l'incidence annuelle signalée et l'incidence annuelle prévue de la PFA chez les enfants de moins de 15 ans. En utilisant un test de probabilité de Poisson, nous avons testé la significativité de la corrélation entre les augmentations annuelles imprévues des cas de PFA et l’émergence du virus Zika. Les données ont été analysées séparément pour chaque territoire et chaque île du Pacifique.

**Environnement local:**

Dans la plupart des îles du Pacifique, les activités de surveillance et d'alerte anticipée des menaces aiguës de santé publique (telles que celle représentée par le virus Zika) sont rendues difficiles par les déficiences des infrastructures de santé, l'insuffisance des ressources humaines et l'isolement géographique des populations.

**Changements significatifs:**

Nous n'avons constaté qu'un seul exemple (Îles Salomon, 2015) d'une augmentation significative des cas signalés de PFA coïncidant avec une émergence du virus Zika.

**Leçons tirées:**

Nous n'avons trouvé aucune preuve concluante permettant d'affirmer que les données systématiquement signalées sur l'incidence de la PFA chez les enfants ont été utiles, dans ces contextes, pour détecter une émergence du virus Zika. Des données complémentaires pourraient être nécessaires en lien avec les populations adultes, qui sont plus susceptibles d'être affectées par le syndrome de Guillain-Barré. Il est également possible que le signalement des PFA soit défaillant dans ces régions certifiées exemptes de poliomyélite.

## Resumen

**Situación:**

La aparición del virus de Zika ha puesto en peligro los sistemas de control del brote en muchos países en riesgo con escasos recursos. Dado que el virus se ha relacionado con el síndrome de Guillain-Barré, los datos rutinarios sobre la incidencia de parálisis flácida aguda (PFA) pueden proporcionar un sistema de advertencia temprana útil para la aparición del virus de Zika.

**Enfoque:**

Se documentaron todos los brotes y casos del virus de Zika en 21 islas y territorios del Pacífico entre los años 2007 y 2015. Se recopiló información de la base de datos de la Iniciativa de Erradicación Mundial de la Poliomielitis sobre la incidencia anual de PFA informada y prevista en niños menores de 15 años. Utilizando una prueba de probabilidad de Poisson, se demostró la importancia de los aumentos inesperados de PFA en los años correlacionados con la aparición del virus de Zika. Se analizaron los datos por separado por cada país y territorio de las Islas del Pacífico.

**Marco regional:**

En la mayoría de los países de las Islas del Pacífico, el control de aviso temprano para amenazas sanitarias públicas agudas como el virus de Zika se ve obstaculizado por una infraestructura sanitaria pobre, escasos recursos humanos y aislamiento geográfico.

**Cambios importantes:**

Únicamente se encontró un ejemplo (Islas Salomón en 2015) de un gran aumento en los casos de PFA informada que se correlacionaron con la aparición del virus de Zika.

**Lecciones aprendidas:**

No se encontraron pruebas concluyentes de que los datos de incidencia de PFA informada de forma rutinaria en niños fueran útiles para detectar la aparición del virus de Zika en esta región. Es posible que se necesiten más pruebas de la población adulta, ya que esta tiene más probabilidades de ser afectada por el síndrome de Guillain-Barré. La notificación de casos de PFA puede ser deficiente en algunas regiones sin incidencias de poliomielitis.

## ملخص

**المشكلة:**

شكل ظهور فيروس زيكا تحديًا لأنظمة الرصد المنتشرة في العديد من البلدان منخفضة الموارد والمعرضة للخطر. وحيث إن الفيروس يرتبط بمتلازمة غيان-باريه، فقد توفر البيانات الروتينية المتعلقة بالإصابة بشلل الرخو الحاد (AFP) نظام تحذير مفيد ومبكر فيما يتعلق بتفشي فيروس زيكا.

**الأسلوب:**

لقد قمنا بتوثيق جميع حالات تفشي فيروس زيكا في 21 إقليمًا وجزيرة من جزر المحيط الهادئ للسنوات من 2007 وحتى 2015. وقمنا باستخراج البيانات من قاعدة البيانات الخاصة بالمبادرة العالمية لاستئصال شلل الأطفال فيما يتعلق بحالات الإصابة بالشلل الرخو الحاد (AFP) سنويًا بين الأطفال دون سن 15 عامًا. وباستخدام اختبار بواسون للاحتمالات، قمنا باختبار أهمية الزيادات غير المتوقعة في حالات الإصابة بالشلل الرخو الحاد (AFP) في السنوات المرتبطة بتفشي فيروس زيكا. وتم تحليل البيانات بشكلٍ منفصل لكل إقليم ودولة من دول جزر المحيط الهادئ.

**المواقع المحلية:**

في أغلب دول جزر المحيط الهادئ، تتم عرقلة رصد التحذيرات المبكرة لتهديدات الصحة العامة الخطرة مثل زيكا فيروس من قبل البنية الأساسية الصحية السيئة، وعدم كفاية الموارد البشرية، والعزل الجغرافي.

**التغيّرات ذات الصلة:**

لم يتم العثور إلا على مثال واحد (جزر سليمان سنة 2015) من الزيادة الواضحة في حالات الإصابة بالشلل الرخو الحاد (AFP) المبلغ عنها المرتبطة بتفشي فيروس زيكا.

**الدروس المستفادة:**

لم نعثر على دليل قاطع يشير إلى أن البيانات المتعلقة بحالات الإصابة بالشلل الرخو الحاد (AFP) في الأطفال المبلغ عنها بشكلٍ روتيني كانت مفيدة في الكشف عن تفشي فيروس زيكا في هذه البيئة. قد يلزم وجود المزيد من الأدلة من القطاعات السكانية من البالغين الأكثر عرضة للتأثر بمتلازمة غيان-باريه. قد يكون الإبلاغ عن الإصابة بالشلل الرخو الحاد (AFP) ضعيفًا في المناطق المصنفة على أنها خالية من شلل الأطفال.

## 摘要

**问题:**

寨卡病毒的出现对许多存在感染风险且资源匮乏国家的疫情监测系统提出了挑战。 由于该病毒与格林 - 巴利综合征有关，有关急性弛缓性麻痹 (AFP) 发病率的常规数据可提供一个有效的监测寨卡病毒出现的早期预警系统。

**方法:**

我们记录了 2007 年至 2015 年间 21 个太平洋岛屿及地区的所有寨卡病毒疫情和病例，并从全球根除脊髓灰质炎行动数据库中提取了 15 岁以下儿童年 AFP 发病率的报告值和预测值数据。 我们利用泊松概率测试 (Poisson probability test) 对寨卡病毒出现年份里 AFP 发病率的意外增加进行了显著性测试。 每个太平洋岛屿国家和地区的数据进行了独立分析。

**当地状况:**

在大多数太平洋岛屿国家，对诸如寨卡病毒等突发公共卫生威胁的早期预警监测受到薄弱的卫生基础设施、人力资源不足以及地域隔离的制约。

**相关变化:**

在报告的寨卡病毒出现相关 AFP 病例中，仅发现一例 AFP 发病率显著增加的病例（2015 年，所罗门群岛）。

**经验教训:**

我们没有发现明确证据表明定期报告儿童 AFP 发病率数据有助于检测该地区寨卡病毒的出现。 我们可能需要从更有可能感染格林 - 巴利综合征的成年人口那里获得更多证据。 AFP 报告在被证实无脊髓灰质炎的地区可能作用有限。

## Резюме

**Проблема:**

Появление вируса Зика — важный предмет систем эпидемиологического надзора во многих бедных странах риска. Так как вирус был ассоциирован с синдромом Гийена — Барре, регулярно собираемые данные по заболеваемости остро развивающимся периферическим параличом (ОРПП) могут служить для полезной системы раннего предупреждения по вирусу Зика.

**Подход:**

Нами задокументированы все вспышки и случаи заражения вирусом Зика на 21 острове и территории Океании в период с 2007 по 2015 год. Сведения были получены из базы данных Глобальной инициативы по ликвидации полиомиелита и касались сообщаемой и прогнозируемой ежегодной заболеваемости ОРПП у несовершеннолетних моложе 15 лет. С помощью критерия Пуассона нами была проверена статистическая значимость неожиданных повышений частоты ОРПП по годам, коррелирующая с заболеваемостью вирусом Зика. Данные были проанализированы отдельно для каждого острова и территории Океании.

**Местные условия:**

В большинстве стран Океании ранний предупреждающий надзор по таким важным угрозам для здоровья, как вирус Зика, был осложнен плохой инфраструктурой здравоохранения, недостаточным количеством персонала и географической изоляцией.

**Осуществленные перемены:**

Был только один пример (Соломоновы острова, 2015) статистически значимого увеличения зарегистрированных случаев ОРПП, коррелирующих с заболеваемостью вирусом Зика.

**Выводы:**

Нами не было найдено доказательств, позволяющих заключить, что регулярно сообщаемые данные об ОРПП у несовершеннолетних могут быть полезны для определения появления вируса Зика в этих условиях. Возможно, требуется больше данных из взрослых групп населения, которые затрагиваются синдромом Гийена — Барре с большей вероятностью. Сообщений об ОРПП может быть также недостаточно в регионах, для которых подтверждено отсутствие полиомиелита.

## Introduction

In February 2016, in response to outbreaks in several Pacific and South American countries,^1^ Zika virus was declared a Public Health Emergency of International Health Concern by the World Health Organization (WHO).^2^ This was based on increasing evidence that Zika virus infection may be associated with congenital malformations and autoimmune neurological diseases, including microcephaly, cranial nerve dysfunction and Guillain–Barré syndrome.^1^^,^^2^

The emergence of the Zika virus has challenged basic outbreak surveillance systems in many at-risk, low-resource countries. Zika virus surveillance strategies need to be convenient, timely and cost-effective, ideally using routinely collected information. Data on the incidence of acute flaccid paralysis (AFP) in children younger than 15 years are routinely collected for polio surveillance by 177 of the 194 WHO Member States as part of the Global Polio Eradication Initiative. The most common cause of AFP is Guillain–Barré syndrome.^3^ As the syndrome has been associated with Zika virus infection, increases in the incidence of AFP – routinely reported to the Global Polio Eradication Initiative – might provide a useful early warning for Zika virus outbreaks in resource-constrained settings. We analysed data from the Pacific Islands to test this hypothesis. 

## Local setting

The Pacific Islands, which fall within WHO’s Western Pacific Region, are home to several of the world’s smallest, least developed and most isolated populations. The collective population of these islands (excluding New Zealand) is approximately 11.4 million people, of whom 8.2 million reside in Papua New Guinea and the rest are dispersed over the thousands of islands and atolls that make up the other 20 Pacific Island countries and territories. Most islands’ health authorities rely on simple syndromic surveillance and ad hoc event reporting by clinicians for disease outbreak detection. Their ability to enhance early warning surveillance in response to acute public health threats such as Zika virus is hampered by limited coverage and sensitivity of existing early warning surveillance; limited capacity to investigate outbreaks; geographic isolation and widely dispersed populations; poor communication infrastructure; and inadequately resourced health systems.

## Approach

We documented all Zika virus outbreaks and cases in the Pacific Islands for the years 2007–2015 and tested the significance of unexpected increases in AFP in years that correlated with Zika virus emergence.

We obtained data on Zika virus outbreaks in the 21 Pacific Island countries and territories from published and unpublished information. For published information, we performed a literature search using the search terms “Zika” and “Zika virus” in the PubMed database; the identified papers were reviewed for relevance to the Pacific Islands. Unpublished event-relevant information was extracted from WHO’s weekly Pacific Surveillance Syndromic Reports^4^ and from PacNet listserv posts.^5^ PacNet is the email-based outbreak notification and discussion forum of the Pacific Public Health Surveillance Network. Data extracted included the start and end dates of events, and the reported number of suspected and confirmed cases. To verify information extracted from unpublished sources we consulted staff at WHO’s Division of Pacific Technical Support in Fiji. We also collected information on Zika outbreaks and cases from January to November 2016 to provide a complete and up-to-date picture of Zika activity in the study area.

AFP surveillance for polio eradication purposes requires health workers to promptly report and investigate identified cases of AFP, including the results of testing for wild polio virus. We extracted data on the expected and reported annual incidence of AFP in children younger than 15 years for Pacific Island countries and territories from the Global Polio Eradication Initiative’s surveillance database.^6^ Then we compared these data with documented Zika virus outbreaks to identify space–time correlations.

We used the following Poisson probability formula to test the significance (at *P* ≤ 0.05) of unexpected increases in the incidence of AFP in children in the years when Zika virus emerged in each Pacific Island country or territory:

$$P(X=x)=\frac{e^{-\lambda }\lambda^{x}}{x!}$$ (1)

Where *P* is the probability; *X* is probability mass function; *x* is the number of events in a specified time period; e is the mathematical constant (Euler’s number, approximately 2.72); and *λ* is the expected number of events in the specified time period. 

## Zika virus cases

The first human outbreak of Zika virus was documented in the Pacific Islands in Yap State, Federated States of Micronesia in April 2007.^7^ The investigators identified 185 suspected cases, of which 49 were confirmed. No further Zika cases were detected in the Pacific Islands until October 2013, when an outbreak of 383 confirmed cases occurred in French Polynesia.^8^^,^^9^ Given travel pathways and close geographical and cultural ties, the outbreak in French Polynesia was suspected to have been the source of subsequent outbreaks on Easter Island (January–May 2014; 51 confirmed cases),^10^ New Caledonia (January–August 2014, more than 1400 confirmed cases^11^ and January–May 2015, 82 confirmed cases)^12^ and Cook Islands (February–May 2014; 54 confirmed cases).^4^ In 2015 two other countries reported emergence and autochthonous transmission of Zika virus: Solomon Islands (February–May 2015; 5 confirmed cases) and Samoa (September 2015 to May 2016; 24 confirmed cases). In 2015, sporadic (non-autochthonous) Zika cases were reported from Vanuatu (one case confirmed, April 2015) and Fiji (at least 15 cases, between August 2015 and June 2016).^4^^,^^5^

### 2016 update

In 2016 (outside the analysis window) three other Pacific Island countries and territories and Kosrae State, in the Federated States of Micronesia (on which Zika had previously not been detected) reported autochthonous transmission: Tonga (January–April 2016; two confirmed cases), American Samoa (February 2016 and ongoing; 52 confirmed cases as at 3 November 2016), Marshall Islands (February–April 2016; two confirmed cases) and Kosrae State (February 2016 and ongoing; 23 confirmed cases as at 2 November 2016). In November 2016, Palau reported a single confirmed Zika case.^2^^,^^5^

In addition, in March 2016 Papua New Guinea reported that six cases of Zika virus infection had been confirmed through retrospective testing of samples collected in May 2015 (one case), December 2015 (two cases) and February 2016 (three cases), indicating low levels of Zika virus transmission within the country.^13^

## AFP cases

Based on Global Polio Eradication Initiative predictions, the total expected annual number of AFP cases for the year 2015 was 36 (26 for Papua New Guinea and 10 for the other 20 Pacific Island countries and territories). The aggregated number of AFP cases among children reported in each year were 38 (2007), 35 (2008), 30 (2009), 37 (2010), 26 (2011), 14 (2012), 18 (2013), 27 (2014) and 41 (2015; Table 1).

**Table 1:** Reported Zika virus outbreaks in the Pacific Islands and acute flaccid paralysis cases in children aged < 15 years reported to the Global Polio Eradication Initiative, by country or territory and year, 2007–2015

Pacific Island countries and territories	Population in 2016^a^	Annual no. of AFP cases expected^b^	No. of reported cases of AFP^b^ (Zika virus outbreaks: dates; no. of confirmed cases^c^)								
			2007	2008	2009	2010	2011	2012	2013	2014	2015
American Samoa	56 400	< 1	0 (0)	0 (0)	0 (0)	0 (0)	0 (0)	0 (0)	0 (0)	0 (0)	0 (0)
Cook Islands	15 200	< 1	0 (0)	0 (0)	0 (0)	0 (0)	0 (0)	0 (0)	0 (0)	0 (Feb–May; 54)	0 (0)
Fiji	880 400	3	7 (0)	4 (0)	9 (0)	5 (0)	6 (0)	4 (0)	7 (0)	8 (0)	4 (Aug; 2)
French Polynesia	273 800	< 1	1 (0)	0 (0)	0 (0)	0 (0)	0 (0)	0 (0)	0 (Oct–Dec; 227)	0 (Jan–Jul; 156)	0 (0)
Guam	169 500	< 1	0 (0)	0 (0)	0 (0)	0 (0)	0 (0)	0 (0)	0 (0)	0 (0)	0 (0)
Kiribati	113 000	< 1	0 (0)	0 (0)	0 (0)	0 (0)	0 (0)	0 (0)	0 (0)	0 (0)	0 (0)
Marshall Islands	55 000	< 1	0 (0)	0 (0)	0 (0)	0 (0)	0 (0)	0 (0)	0 (0)	0 (0)	0 (0)
Micronesia (Federated States of)	104 600	< 1	0 (Apr–Jun; 49)	0 (0)	0 (0)	0 (0)	0 (0)	0 (0)	0 (0)	0 (0)	0 (0)
Nauru	10 800	< 1	0 (0)	0 (0)	0 (0)	0 (0)	0 (0)	0 (0)	0 (0)	0 (0)	0 (0)
New Caledonia	277 000	< 1	1 (0)	1 (0)	3 (0)	1 (0)	0 (0)	0 (0)	0 (0)	0 (Jan–Aug; > 1 400^d^)	0 (Jan–May; 82)
Niue	1 600	< 1	0 (0)	0 (0)	0 (0)	0 (0)	0 (0)	0 (0)	0 (0)	0 (0)	0 (0)
Northern Marianas Islands (Commonwealth of)	55 700	< 1	0 (0)	1 (0)	0 (0)	0 (0)	0 (0)	0 (0)	0 (0)	0 (0)	0 (0)
Palau	17 800	< 1	0 (0)	0 (0)	0 (0)	0 (0)	0 (0)	0 (0)	0 (0)	0 (0)	0 (0)
Papua New Guinea	8 151 300	26	25 (0)	12 (0)	24 (0)	17 (0)	10 (0)	10 (0)	18 (0)	12 (0)	27 (May & Dec; 3^e^)
Samoa	194 000	< 1	1 (0)	0 (0)	1 (0)	0 (0)	0 (0)	0 (0)	0 (0)	0 (0)	0 (Sep–Dec; ~3)
Solomon Islands^f^	651 700	2	3 (0)	3 (0)	3 (0)	7 (0)	3 (0)	0 (0)	1 (0)	6 (0)	9 (Feb–May; 5)
Tokelau	1 400	< 1	0 (0)	0 (0)	0 (0)	0 (0)	0 (0)	0 (0)	0 (0)	0 (0)	0 (0)
Tonga	100 600	< 1	0 (0)	0 (0)	1 (0)	0 (0)	0 (0)	0 (0)	0 (0)	0 (0)	0 (0)
Tuvalu	10 100	< 1	0 (0)	0 (0)	0 (0)	0 (0)	0 (0)	0 (0)	0 (0)	0 (0)	0 (0)
Vanuatu	279 700	1	0 (0)	1 (0)	0 (0)	0 (0)	0 (0)	0 (0)	0 (0)	1 (0)	1 (Apr; 1)
Wallis and Futuna	11 800	< 1	0 (0)	0 (0)	0 (0)	0 (0)	0 (0)	0 (0)	0 (0)	0 (0)	0 (0)
Total	11 441 400	36	38 (49)	35 (0)	30 (0)	37 (0)	26 (0)	14 (0)	18 (227)	27 (1 700)	41 (96)

AFP: acute flaccid paralysis; GPEI: Global Polio Eradication Initiative.

^a^ Estimated populations were obtained from the Secretariat of the Pacific Community database (https://prism.spc.int/regional-data-and-tools/population-statistics/169-pacific-island-populations).

^b^ Based on predictions of GPEI for 2015. Expected number of cases were the same for all reported preceding years except for Papua New Guinea in 2007 (25 cases). Data on AFP were from the Global Polio Eradication Initiative database.^6^

^c^ Data on Zika virus outbreaks (shown in bold) were extracted from published literature, WHO’s weekly Pacific Syndromic Surveillance Reports and posts to the Pacific Public Health Surveillance Network’s listserv.^5^ Criteria for suspected and confirmed Zika virus cases vary. Suspected case definitions typically require a patient to have a rash or fever with any of the following: pain behind the eyes, conjunctivitis, body aches or oedema of hands or feet. Confirmation of suspected cases require a positive result from a laboratory test, either polymerase chain reaction assay or a serological test, such as immunoglobulin (Ig)M or serial IgG assay.

^d^ Exact number of cases was not available.

^e^ Zika virus infection was confirmed through retrospective testing of samples collected between July 2014 and March 2016.

^f^ Correlation between higher than expected number of acute flaccid paralysis cases and Zika virus emergence was significant for the Solomon Islands in 2015 (*P* ≤ 0.001).

Analysis of individual Pacific Island countries and territories found only one example – the Solomon Islands in 2015 – where a statistically significant increase in reported AFP cases correlated with the emergence of Zika virus (*P* ≤ 0.001). From February to May 2015, there were five confirmed cases of Zika virus infection out of 324 suspected cases in a population of about 651 700 people. In that year, nine cases of AFP were reported compared with the expected number of two. None of the seven other countries and territories reporting Zika virus cases from 2007 to 2015 (Cook Islands, Federated States of Micronesia, Fiji, French Polynesia, New Caledonia, Samoa and Vanuatu) showed significant rises in reported cases of AFP associated with emergence of Zika virus.

The significant increase in AFP cases in the Solomon Islands may be an indication of the usefulness of AFP detection for signalling the appearance of a Zika virus outbreak. Alternatively, it may reflect an increased vigilance of public health surveillance following the major tropical cyclone Raquel, which affected the Solomon Islands in July 2015, or it may just be an anomaly.

## Lessons learnt

Recommendations to enhance surveillance for Zika virus in at-risk countries have included improving surveillance for Guillain–Barré syndrome via the existing surveillance systems for AFP used by polio eradication programmes.^14^ Our analysis, however, did not provide sufficient evidence that analysis of AFP incidence data collected for children provide a useful surveillance strategy to detect Zika virus emergence in this setting (Box 1).

Box 1 **Summary of main lessons learnt**

- Routinely reported data on acute flaccid paralysis (AFP) incidence in children were insufficient for the identification of Zika virus emergence in Pacific Island countries and territories.
- Data from adults, who are more affected by Guillain-Barré syndrome, may confirm whether AFP incidence is a suitable early warning surveillance strategy for emergence of Zika virus in low-resource settings.
- More evidence is needed that AFP reporting requirements are being met in remote areas and in regions certified as polio free.

Populations in Pacific Island countries are small and it is likely that the capacity to conduct and ensure compliance with AFP reporting requirements varies. This may be influenced by a lack of awareness of polio surveillance and associated AFP reporting, given that the last indigenous case of polio virus in the Western Pacific Region was reported in Cambodia in 1997, and the Region has been certified as polio free since 2000.^15^ Small population sizes also means that the expected incidences of AFP in individual countries and territories are very low (often < 1 case) and therefore statistical power may be lacking. More evidence is needed to determine whether the case detection of AFP is compromised in remote areas and in regions certified as polio free.

It should be noted that the Global Polio Eradication Initiative’s AFP surveillance targets paediatric populations, who are less likely than adults to be affected by Guillain–Barré syndrome.^3^ Data that include adult age groups (which is not currently routine practice) may provide better evidence to determine whether AFP surveillance offers a suitable strategy for Zika virus early warning in low-resource settings, such as the Pacific Islands.

## References

[1] Zika virus, microcephaly and Guillain–Barre syndrome: situation report, 13 October 2016. Geneva: World Health Organization; 2016. Available from: http://apps.who.int/iris/bitstream/10665/250512/1/zikasitrep13Oct16-eng.pdf?ua=1[cited 2016 Oct 20].

[2] WHO statement on the first meeting of the International Health Regulations (2005) (IHR 2005) Emergency Committee on Zika virus and observed increase in neurological disorders and neonatal malformations. Geneva: World Health Organization; 2016. Available from: http://www.who.int/mediacentre/news/statements/2016/1st-emergency-committee-zika/en/ [cited 2016 Feb 9].

[3] Sejvar JJ, Baughman AL, Wise M, Morgan OW. Population incidence of Guillain-Barré syndrome: a systematic review and meta-analysis. Neuroepidemiology. 2011;36(2):123–33. 10.1159/000324710 21422765 PMC5703046

[4] Weekly pacific syndromic surveillance report [Internet]. Suva: World Health Organization Division of Pacific Technical Support; 2016. Available from: http://www.wpro.who.int/southpacific/programmes/communicable_diseases/disease_surveillance_response/page/en/index2.html[cited 2016 Nov 16].

[5] PacNet [Internet]. Noumea: Pacific Public Health Surveillance Network; 2016. Available from: http://www.pphsn.net/services/PacNet/intro.htm[cited 2016 Nov 16].

[6] Polio now [Internet]. Geneva: The Global Polio Eradication Initiative; 2016. Available from: http://polioeradication.org/polio-today/polio-now/ [cited 2016 Nov 21].

[7] Duffy MR, Chen T-H, Hancock WT, Powers AM, Kool JL, Lanciotti RS, et al. Zika virus outbreak on Yap Island, Federated States of Micronesia. N Engl J Med. 2009 6 11;360(24):2536–43. 10.1056/NEJMoa0805715 19516034

[8] Cao-Lormeau V-M, Roche C, Teissier A, Robin E, Berry A-L, Mallet H-P, et al. Zika virus, French Polynesia, south Pacific, 2013. Emerg Infect Dis. 2014 6;20(6):1085–6. 10.3201/eid2006.140138 24856001 PMC4036769

[9] Kucharski AJ, Funk S, Eggo RM, Mallet H-P, Edmunds WJ, Nilles EJ. Transmission dynamics of Zika virus in island populations: a modelling analysis of the 2013–14 French Polynesia outbreak. PLoS Negl Trop Dis. 2016 5;10(5):e0004726. 10.1371/journal.pntd.0004726 27186984 PMC4871342

[10] Tognarelli J, Ulloa S, Villagra E, Lagos J, Aguayo C, Fasce R, et al. A report on the outbreak of Zika virus on Easter Island, South Pacific, 2014. Arch Virol. 2016 3;161(3):665–8. 10.1007/s00705-015-2695-5 26611910

[11] Dupont-Rouzeyrol M, O’Connor O, Calvez E, Daurès M, John M, Grangeon J-P, et al. Co-infection with Zika and dengue viruses in 2 patients, New Caledonia, 2014. Emerg Infect Dis. 2015 2;21(2):381–2. 10.3201/eid2102.141553 25625687 PMC4313662

[12] Situation actuelle en Nouvelle Calédonie, Nouméa 2015. Noumea: Government of New Caledonia; 2015. Available from: http://www.dass.gouv.nc/portal/page/portal/dass/observatoire_sante/veille_sanitaire/Zika[cited 2016 Feb 15]. French.

[13] Zika virus infection – Papua New Guinea [Disease Outbreak News 22 April 2016 [Internet]. Geneva: World Health Organization; 2016. Available from: http://www.who.int/csr/don/22-april-2016-zika-png/en/ [cited 2016 Nov 22].

[14] Kandel N, Lamichhane J, Tangermann RH, Rodier GR. Detecting Guillain-Barré syndrome caused by Zika virus using systems developed for polio surveillance. Bull World Health Organ. 2016 9 1;94(9):705–8. 10.2471/BLT.16.171504 27708476 PMC5034644

[15] Health topics: poliomyelitis [Internet]. Manila: World Health Organization Regional Office for the Western Pacific; 2016. Available from: http://www.wpro.who.int/topics/poliomyelitis/en/ [cited 2016 Nov 16].

